# Modified Early Warning Score (MEWS) Identifies Critical Illness among Ward Patients in a Resource Restricted Setting in Kampala, Uganda: A Prospective Observational Study

**DOI:** 10.1371/journal.pone.0151408

**Published:** 2016-03-17

**Authors:** Rebecca Kruisselbrink, Arthur Kwizera, Mark Crowther, Alison Fox-Robichaud, Timothy O'Shea, Jane Nakibuuka, Isaac Ssinabulya, Joan Nalyazi, Ashley Bonner, Tahira Devji, Jeffrey Wong, Deborah Cook

**Affiliations:** 1 Department of Medicine, McMaster University, Hamilton, Ontario, Canada; 2 Department of Anesthesia and Critical Care, Makerere University, Kampala, Uganda; 3 Department of Critical Care, McMaster University, Hamilton, Ontario, Canada; 4 Department of Medicine, Mulago Hospital, Kampala, Uganda; 5 Department of Medicine, Makerere University, Kampala, Uganda; 6 Department of Biostatistics, McMaster University, Hamilton, Ontario, Canada; 7 Department of Health Research Methodology, McMaster University, Hamilton, Ontario, Canada; 8 Department of Medicine, University of Toronto, Toronto, Ontario, Canada; University of Pittsburgh, UNITED STATES

## Abstract

**Introduction:**

Providing optimal critical care in developing countries is limited by lack of recognition of critical illness and lack of essential resources. The Modified Early Warning Score (MEWS), based on physiological parameters, is validated in adult medical and surgical patients as a predictor of mortality. The objective of this study performed in Uganda was to determine the prevalence of critical illness on the wards as defined by the MEWS, to evaluate the MEWS as a predictor of death, and to describe additional risk factors for mortality.

**Methods:**

We conducted a prospective observational study at Mulago National Referral Teaching Hospital in Uganda. We included medical and surgical ward patients over 18 years old, excluding patients discharged the day of enrolment, obstetrical patients, and patients who self-discharged prior to study completion. Over a 72-hour study period, we collected demographic and vital signs, and calculated MEWS; at 7-days we measured outcomes. Patients discharged prior to 7 days were assumed to be alive at study completion. Descriptive and inferential statistical analyses were performed.

**Results:**

Of 452 patients, the median age was 40.5 (IQR 29–54) years, 53.3% were male, 24.3% were HIV positive, and 45.1% had medical diagnoses. MEWS ranged from 0 to 9, with higher scores representing hemodynamic instability. The median MEWS was 2 [IQR 1–3] and the median length of hospital stay was 9 days [IQR 4–24]. In-hospital mortality at 7-days was 5.5%; 41.4% of patients were discharged and 53.1% remained on the ward. Mortality was independently associated with medical admission (OR: 7.17; 95% CI: 2.064–24.930; p = 0.002) and the MEWS ≥ 5 (OR: 5.82; 95% CI: 2.420–13.987; p<0.0001) in the multivariable analysis.

**Conclusion:**

There is a significant burden of critical illness at Mulago Hospital, Uganda. Implementation of the MEWS could provide a useful triage tool to identify patients at greatest risk of death. Future research should include refinement of MEWS for low-resource settings, and development of appropriate interventions for patients identified to be at high risk of death based on early warning scores.

## Introduction

Critical illness is a substantial burden in developing countries, [1] contributed to by high rates of malnutrition, infection including HIV/AIDs, trauma, and maternal morbidity.[2] Reliable epidemiological data on critical illness in low-resource settings are scarce,[3,4] compared to conditions such as tuberculosis, HIV, or cancer, for which estimates of global disease burden are available from multiple sources.[5]

Documenting the burden of critical illness in low-resource settings is challenging; it is difficult to measure precisely as syndromes such as sepsis and multi-organ failure are not captured by a diagnostic test [6,7] and illness severity measures are frequently unavailable. Critically ill patients are often cared for on the wards due to the paucity of Intensive Care Unit (ICU) beds. Fatality rates are high, limiting prevalence data.[8]

Providing optimal critical care in low-resource settings is constrained by lack of essential medication, equipment, and clinicians. [9,10] Anesthesiologists surveyed in sub-Saharan African hospitals revealed that the Surviving Sepsis Campaign Guidelines could be implemented in entirety in only 1.4% of sites. [11]

A feasible, low-cost method of rapidly identifying patients requiring critical care is needed. Early warning scores utilize physiological, easy-to-measure parameters such as vital signs and level of consciousness to identify critical illness, facilitate early intervention, and predict mortality. [12,13] In a seminal study of the Modified Early Warning Score (MEWS) (Table 1) applied to acute medical admissions, Subbe et al showed that having a MEWS of 5 or greater was associated with increased risk of death (OR 5.4, 95%CI 2.8–10.7) and ICU admission. [12] In the first-world setting, early warning scores have been utilized to achieve earlier interventions, [12–14] but broader application is possible in low-resource settings because of their simplicity. Several variants, including the MEWS, have been validated in African settings. [15–18]

**Table 1:** The Modified Early Warning Score. This table shows the vital sign parameters comprising the Modified Early Warning Score. Adapted from Subbe et al, 2001 [12].

	3	2	1	0	1	2	3
**Systolic blood pressure (mmHg)**	< 70	71–80	81–100	101–199		> 200	
**Pulse rate (beats per minute)**		< 40	41–50	51–100	101–110	111–129	> 130
**Respiratory rate (breaths per minute)**		< 9		9–14	15–20	21–29	>30
**Temperature (**^**o**^**C)**		<35		35–38.4		>38.5	
**AVPU score**				Alert	Reaction to voice	Reaction to pain	Unresponsive
**AVPU: A, alert; V, responding to voice, P, responding to pain; U, unresponsive.**							

The primary objective of this study was to determine the prevalence of critical illness in the Mulago National Referral Hospital (MNRH) using the MEWS as a measure of illness severity. Secondary objectives were to evaluate the utility of the MEWS as a predictor of 7-day in-hospital mortality, and to describe additional risk factors for mortality among patients admitted to a tertiary-level African government hospital.

## Materials and Methods

### Patients

We conducted a prospective observational study of all patients on the adult medical and surgical wards of Mulago Hospital over a 10-day period in February 2013. Patients were enrolled during one of three consecutive study days, including patients admitted from the emergency department as well as transfers from the ICU or other private and government hospitals. We excluded patients less than 18 years of age, patients discharged the same day, patients in whom non-invasive blood pressure measurement could not be obtained, and obstetrical patients who have distinct, well-characterized risk factors for mortality, in whom MEWS has not been validated [19] and who are monitored in a separate high-dependency unit (HDU).

### Study setting

Uganda, an East African nation with a population of 36 million, has a national critical care capacity of 33 beds, two thirds of which are in the capital city of Kampala. [20] Mulago Hospital is Uganda’s largest government hospital, with 1500 beds, 30,000 deliveries annually, over 150 adult admissions daily, and more than 100 surgical cases per day. Critical care capacity includes 12 ICU beds with 4 functional ventilators and two small HDUs on the neurosurgical and obstetrical wards. [20] Mortality data are limited, but published and unpublished sources indicate that across all departments, mortality is high, particularly among patients identified as being critically ill, septic, or in shock. Hospital statistics from 2008 indicate in-hospital mortality of 15.4% on the medical wards. [21] In-hospital mortality among 380 patients with severe sepsis was 23.7% in a 2008 prospective observational study of sepsis management and outcomes. [21]

### Data collection

Data were collected by one of a 20-member team of study personnel comprised of physicians, medical students, and nursing students. Each member was trained on the case report form, piloted the data collection and had calibration feedback as necessary. Starting on the day of enrollment, each patient had the following collected: current vital signs as recorded by the study team, demographic data including age, sex, admission diagnosis, assigned ward, diagnostic, monitoring and therapeutic interventions, and duration of hospital stay. Documentation of vital signs during the previous 48 hours on the wards and in the emergency department was also recorded, as either present or absent.

### MEWS documentation

MEWS values range from 0 to a maximum of 14; higher scores represent greater hemodynamic instability. Based on Subbe’s study, we defined a MEWS of 5 or greater as identifying a critically ill patient. [12] Study personnel collected all MEWS-related data points for each patient at the time of enrollment to ensure consistency in measurement, and because of the anticipated paucity of vital sign monitoring on the wards. Measured vital signs included heart rate (beats per minute), systolic and diastolic blood pressure (millimeters of mercury), respiratory rate (breaths per minute), axillary temperature (degrees Celsius), and level of consciousness. Level of consciousness was assessed using the Alert, Voice, Pain, Unresponsive (AVPU) score. [12] MEWS were calculated for each patient based on their vital signs at the time of initial assessment by the study personnel. At 7 days, the following outcomes were documented: in-hospital death, discharge, self-discharge from hospital, or alive and on the ward. Patients discharged alive from hospital prior to the 7-day follow up were assumed to be alive at 7 days, when the study was completed.

### Ethics

Ethics approval and permission for waiver of patient consent were obtained from the institutional review board of Mulago Hospital. Anonymized data were entered into an Excel database.

### Data analysis

Descriptive and statistical analyses were performed using SAS software version 9.3. Continuous data are presented as medians with interquartile range (IQR) or means with standard deviation. Categorical data are presented in percentages. Based on previous studies demonstrating the predictive value of MEWS among ward and emergency room patients, threshold scores of 4 and 5 were used. [12,15] Univariable logistic regression models were built to estimate unadjusted odds ratios between each factor and mortality. Multivariable logistic regression analysis using backward stepwise elimination was conducted to identify independent predictors of mortality. A p value of < 0.05 was considered statistically significant.

## Results

Of 530 patients, 62 were excluded due to self-discharge from hospital without complete follow up data, 5 because of incomplete vital signs and inability to calculate a MEWS, and 7 because of missing 7-day outcomes. Overall, 452 patients were included in the analysis (Table 2). Of these, 53.3% were male, 45.1% were medical patients and 18.8% were trauma patients. 24.3% were HIV positive. The median (IQR) age of patients was 40.5 years (29–54); mean age was 42.8, (standard deviation 16.6). Most patients were admitted through the emergency department (53.8%), 30.0% were referred from government units and 16.2% from private hospitals or clinics. Median duration of hospital stay prior to data collection was 9 days [IQR 4–24]. Analysis of baseline features for the group of patients who self-discharged showed no statistically significant differences between groups except for an increase in the proportion of medical patients and the number of HIV-positive patients. The complete comparative analysis is available in table format in Table A in S1 Appendix.

**Table 2:** Baseline characteristics of enrolled patients. * Baseline characteristics of 452 study patients are presented here, as collected at the time of enrollment. Attendants are family and friends of patients who stay with them in hospital to provide them with food, personal care, and transport. They are also responsible for obtaining all prescribed medications and test results.

Feature		N (%)
Age (mean, SD)		42.8, 16.6
Male		241 (53.5)
HIV positive		110 (32.5)
Attendant*		404 (89.4)
Admission source	Casualty/Emergency Department	242 (53.8)
	Government unit outside Mulago	135 (30)
	Private hospital	73 (16.2)
High Dependency Unit (HDU)		4 (0.9)
Admitted due to trauma		85 (18.8)
Admitted to Medical service		204 (45.1)
Admitted to Surgical service	Preoperative	118 (54.1
	Post operative	100 (45.9

* Except where otherwise indicated, all values are given as N (%).

**Fig 1:**
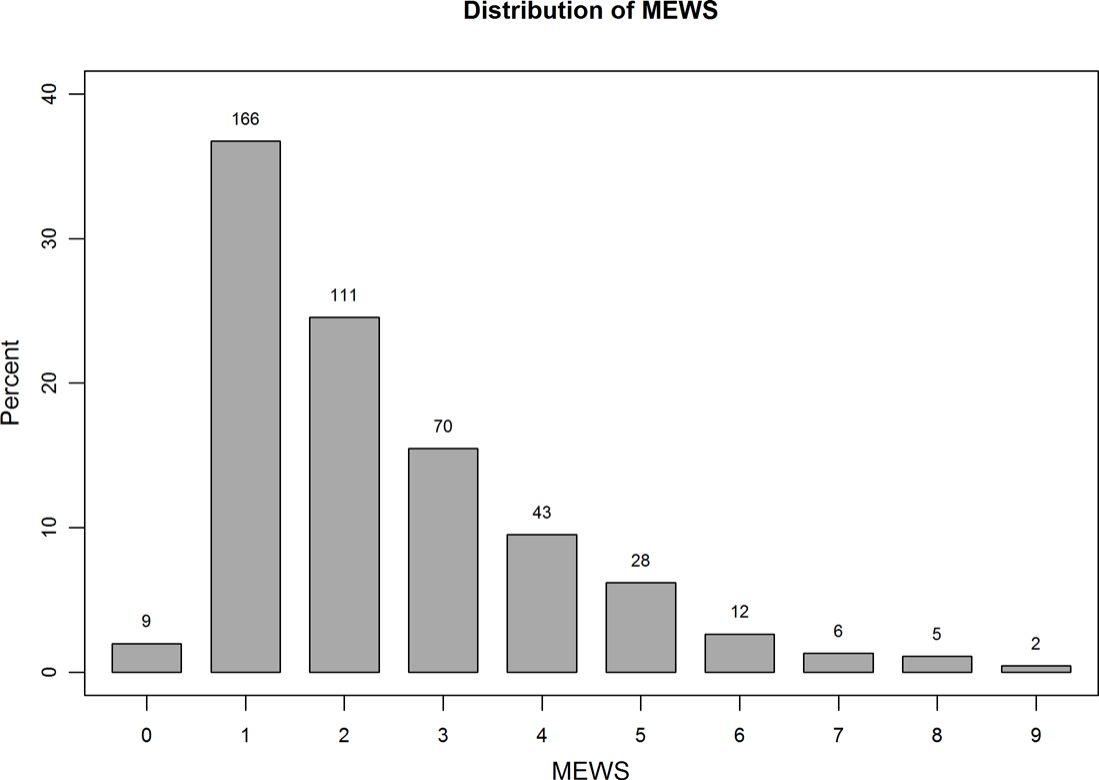
Distribution of MEWS across all patients. Modified Early Warning Scores were calculated for all patients at the time of study enrollment, based on vital signs recorded by research personnel. Scores ranged from 0 to 9, with a median of 2 (IQR 1–3). Mortality increased with higher MEWS.

**Fig 2:**
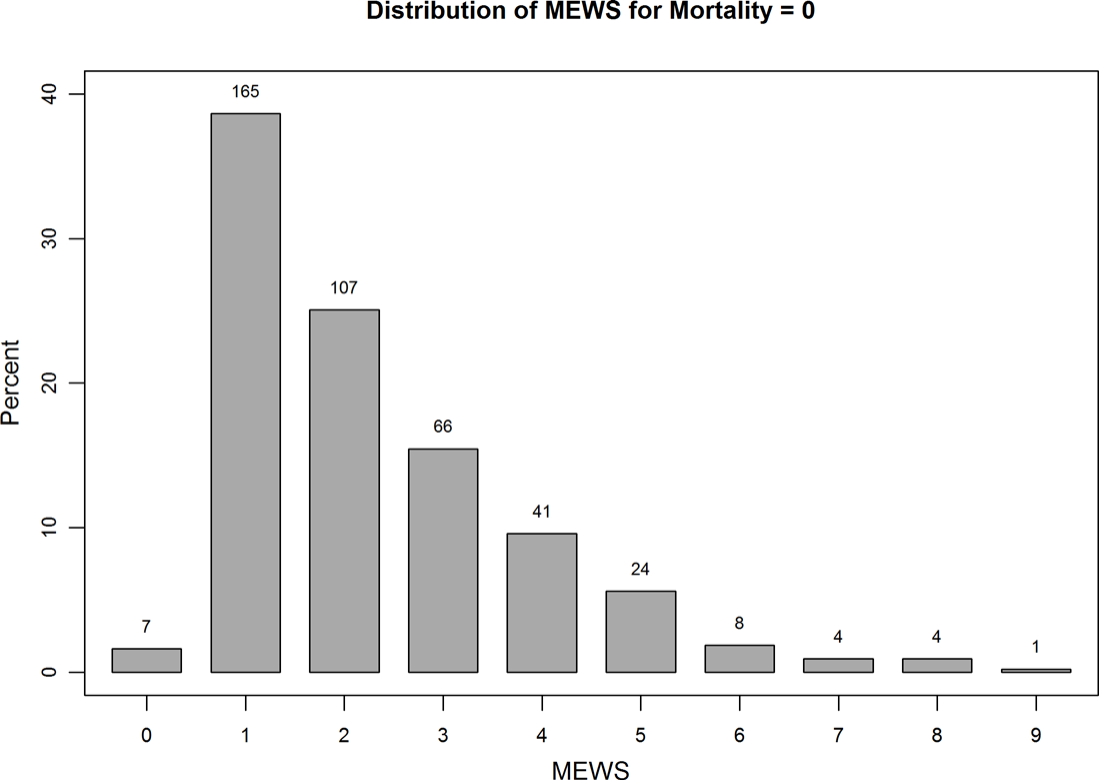
Distribution of MEWS across all patients who survived. Modified Early Warning Scores were calculated for all patients, and the majority of patients who survived had a MEWS of 1, as illustrated in this distribution of MEWS across all surviving patients.

**Fig 3:**
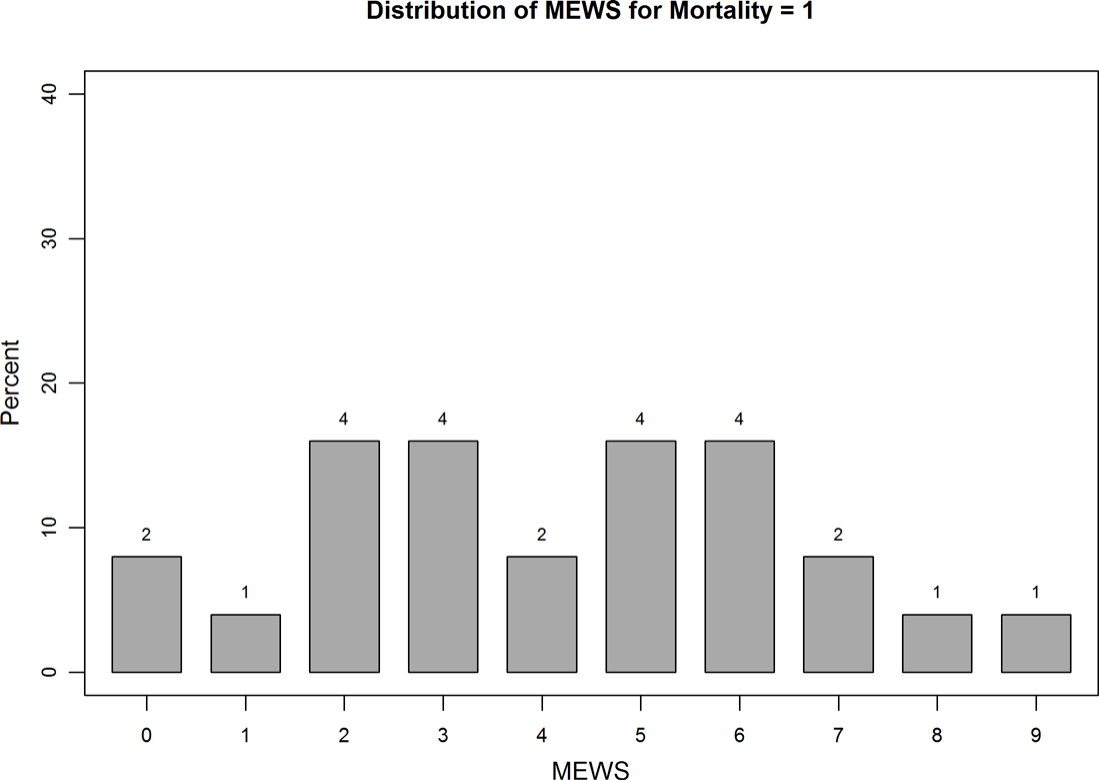
Distribution of MEWS across all patients who died. The distribution of MEWS across patients who did not survive illustrates that MEWS ≥4 was documented in 21.5% of patients; 11.7% of patients had a MEWS ≥5.

MEWS ranged from 0 to 9; (Fig 1), the median score was 2 [IQR 1–3], at a median length of hospital stay of 9 days [IQR 4–24]. Distribution of MEWS across all patients, all patients who survived, and all patients who did not survive are shown separately in Fig 1, Fig 2 and Fig 3. MEWS ≥4 was documented in 21.5% of patients; 11.7% of patients were denoted critically ill, with a MEWS ≥5. No patients within the cohort were admitted to the ICU over the study period. MEWS were higher in medical patients than surgical patients (mean 2.9 versus 1.9, p = 0.001). Although not part of the MEWS, oxygen saturation was measured for each patient; 23 patients (5.1%) had an oxygen delivery device at the time of assessment; 25 patients (5.5%) had a measured saturation of less than 88%.

The overall in-hospital mortality at 7-day follow up was 5.5%; 41.4% of patients were discharged and 53.1% remained on the ward. A MEWS of 4 or greater was a statistically significant predictor of 7-day mortality (OR 5.35, 95% CI 2.35–12.23), as was a MEWS of 5 or greater (OR 8.69, 95% CI 3.72–20.29). Univariate analysis of baseline features associated with mortality showed that in addition to MEWS, HIV positive status (OR 4.23, 95%CI 1.64–10.93), medical admission (OR 9.87, 95%CI 2.91–33.5), and documentation of a systolic blood pressure measured in the emergency room (OR 2.97, 95%CI 1.26–7.04) were associated with an increased risk of death (Table 3).

**Table 3:** Factors Associated with Mortality: Univariate Analysis. MEWS ≥4 and ≥5 were significantly associated with 7-day mortality. Of patients’ baseline features, HIV positive status; admission with a medical diagnosis, and documented blood pressure measurement in the emergency department were also significantly associated with mortality.

		Feature	Odds Ratio (95%CI)	p value
**Demographic**		Age (per year increase)	0.98 (0.95–1.01)	0.103
**features**		Female versus male	0.89 (0.39–2.01)	0.782
		Medical versus surgical	9.87 (2.91–33.5)	0.0002
		Trauma versus non-trauma	0.57 (0.17–1.96)	0.376
		HIV positive	4.23 (1.64–10.93)	0.003
		Duration of hospitalization prior to MEWS (per day increase)	0.97 (0.94–1.01)	0.097
**Patient referral**		ER	0.84 (0.29–2.40)	0.738
		District hospital	0.63 (0.19–2.15)	0.463
		Private hospital	1.0	Reference
**Measured vital signs in**		Heart rate	1.75 (0.78–3.94)	0.179
**ER**		Blood pressure	2.97 (1.26–7.04)	0.013
		Respiratory rate	1.76 (0.64–4.89)	0.276
		Oxygen saturation	1.04 (0.24–4.6)	0.960
		Glasgow coma scale	1.34 (0.58–3.12)	0.492
**Modified early warning score**		MEWS ≥4	5.35 (2.35–12.23)	< 0.0001
		MEWS ≥5	8.69 (3.72–20.29)	< 0.0001

We built a multivariable model using a backward elimination process (variable removal was based on a p = 0.05 threshold), considering MEWS ≥ 5 and the remaining factors that were shown to be significantly associated with mortality in the Univariable analyses. HIV status was excluded from the model because in 25.2% of patients it was not documented. Results are summarized in Table 4. Only medical admission (OR 7.17 [2.064, 24.930], p = 0.002) and MEWS ≥ 5 (OR 5.82 [2.420, 13.987], p<0.0001) were independently associated with mortality; the model had a reliable fit according to a goodness-of-fit assessment of deviance (chi-square = 9.1282; p-value = 0.1041). Using MEWS ≥ 5 alone resulted in an AUROC of 0.692 (95% CI [0.5911, 0.7929]), while adding medical admission status increased the AUROC to 0.804 (95% CI [0.7334, 0.8736]).

**Table 4:** Factors Associated with Mortality: Multivariable Analysis. Of the 4 factors significantly associated with mortality in the univariate analyses, MEWS ≥ 5, medical admission, and systolic blood pressure measurement in the ER were included in the backward stepwise selection procedure in this multivariable analysis. HIV positive status was not included due to the high proportion of missing values. In the final model, medical admission and MEWS ≥ 5were independently associated with mortality.

Variables	Odds Ratio (95% Confidence Interval)	p-value
MEWS ≥ 5	5.82 (2.420, 13.987)	<0.0001
Medical versus surgical	7.17 (2.064, 24.930)	0.002

Our rationale for selecting MEWS ≥ 5 for inclusion in the model is due to its superior specificity and the importance of considering clinician workload in adoption of early warning scores. As shown in Table 5, MEWS cutoff scores of 4 and 5 yielded positive predictive values of 0.15 and 0.23, respectively, in this population. The inverse of the positive predictive value for a given cutoff represents the number of patients that must be evaluated to detect one critical event.[22] For MEWS ≥4, the number needed to evaluate (NNE) was 6.86; for MEWS ≥5, the NNE was 4.42, indicating that for a cutoff of 4, approximately 7 patients need to be evaluated to detect one unstable patient; for a cutoff of 5, fewer than 5 patients need to be evaluated. In resource-constrained environments such as Mulago Hospital, health care providers are limited in number and automated monitoring is scarce. The NNE provides a measure of the nursing workload required to realize the benefit of using the MEWS.

**Table 5:** Calculated indicators for MEWS with cutoffs of 4 and 5. Two-by-two tables showing derivation of prognostic indicators sensitivity, specificity, positive predictive value (PPV), positive likelihood ratio (PLR), and number needed to evaluate (NNE) based on MEWS ≥4 and ≥5, in the study population. *Number needed to evaluate refers to the number of patients required to evaluate to detect one outcome; it is an estimate of the effort-yield of each alert [22].*

	Mortality			Sensitivity	Specificity	Positive Likelihood Ratio (LR+) with 95% CI	Positive Predictive value (PPV)	Number needed to evaluate (NNE)
	Yes	No	Total					
MEWS ≥4	14	82	96	0.5600	0.8080	2.92 [1.958, 4.343]	0.1458	6.86
MEWS < 4	11	345	356					
Totals	25	427	452					
MEWS ≥5	12	41	53	0.4800	0.9040	4.99 [3.029, 8.252]	0.2264	4.42
MEWS < 5	13	386	399					
Totals	25	427	452					

## Discussion

At a large, Sub-Saharan Africa government hospital, we identified that 11.7% of ward patients had critical illness using the MEWS with a cutoff of 5. We found that MEWS was an independent predictor of 7-day in-hospital mortality among mixed medical-surgical ward populations, with an overall 7-day in-hospital mortality of 5.5%. Among patients with MEWS ≥4, mortality was 14.6%, and for MEWS ≥5 mortality was 22.6%. In a multivariate analysis, MEWS and a medical admitting diagnosis were significantly associated with risk of death.

Median length of hospital stay at the time of assessment and MEWS measurement was 9 days [IQR 4–24]. Subbe’s original evaluation of the MEWS among 600 acute medical patients at a British district general hospital showed that only 1.8% had a MEWS ≥5 on their third hospital day, compared to 7.1% at admission. [12] Given the expectation of decreasing acuity (and lower MEWS) throughout the patients’ hospital stay, the higher percentages of critical MEWS among our study population after a median of 9 days in hospital reflect the ongoing burden of critical illness on the wards of Mulago.

Strengths of our study include its prospective, observational design and the heterogeneous sample of patients enrolled. We applied the MEWS across all adult medical and surgical patients in Uganda’s largest government hospital, where the burden of illness, patient volume, and limited resources underscore the immense challenges of delivering critical care. [20] Reports of Mulago’s critically ill patients are emerging but the burden of illness on the wards has not been documented. To our knowledge, this is the first study to utilize the MEWS for this purpose outside of the emergency room, among hospitalized ward patients in Africa. [15–18]

The study has several limitations. Although HIV status was a statistically significant predictor of mortality in the univariate analysis, we did not include it in the multivariable regression model because 25% of patients had no HIV status recorded. We could not ascertain whether these values were unmeasured, measured but not recorded, or recorded but missing from the patient’s file. We evaluated only one major adverse outcome, namely, 7-day mortality. Other studies of early warning scores among hospital inpatients have evaluated additional patient-important outcomes including ICU admission [12,14] and cardiac arrest. [23] Finally, our follow-up period was limited to 7 days. Longer follow up throughout the hospital stay or at one year would have been useful but was not feasible due to resource restrictions; however, 7-day follow-up as a meaningful outcome measure is feasible in this setting, and has been used by other investigators. [24]

An additional limitation is the exclusion of 62 of the 530 patients in our cohort because they self- discharged, or “ran away from hospital”. The reason that patients left prior to discharge was not studied; however, in Ugandan culture, this may have been due to a number of possible reasons. Patients may have left against medical advice, or because they had no attendant to provide their care; they may have gone home to care for children and families, travelled to another hospital for better or less expensive care, or been taken home with their families for palliation. We were unable to follow these patients up outside of hospital, and had no indication of their mortality status at day 7, so we excluded them to avoid misclassification. However, a comparative analysis of the baseline features of 452 analyzed patients, and 62 who self-discharged, is provided in Table A in S1 Appendix (and reveals some interesting findings. The self-discharged patients had a statistically significant higher proportion of medical and HIV-positive patients. Their median MEWS, and proportion of patients with MEWS greater than five, was significantly higher. The group who self-discharged was, therefore, a sicker group of patients and had it been possible to include them, our overall mortality would have likely been higher.

Our overall mortality findings accord with other early warning scores studies reporting 7-day hospital mortality as a primary endpoint. A retrospective review of one million vital signs in an American academic medical center showed that patients with three simultaneous critical vital signs during hospitalization had a mortality of approximately 15% at 7 days and 35% by day 30. [24] A study of 800 medical patients in a South African urban emergency room found in-hospital mortality rates of 16% and 26% among patients with MEWS 3–4 and 26% with MEWS >5, respectively. [15] Assessment of 7-day as well as in-hospital mortality would have been ideal but was not completed due to feasibility constraints of this pilot study. One previous study of septic ward patients at Mulago documented hospital mortality as 24%, similar to our results for patients with MEWS greater than 5. [21]

Among early warning scores in the literature, the MEWS itself has not been shown to be the optimal predictor of mortality. Rather, The VitalPac Early Warning Score (ViEWS), which includes peripheral oxygen saturation and use of supplemental oxygen in addition to pulse rate, systolic blood pressure, respiratory rate, and level of consciousness, is considered to be one of the most suitable predictors of 24-hr mortality in acute medical patients. [16,18,25,26] Using a database of nearly 200,000 observation sets, Prytherch et al demonstrated the superiority of ViEWS to MEWS as well as 33 other early warning scores using the AUROC (area under receiver operating characteristics curve). [16] Our rationale for using the MEWS rather than the ViEWS was grounded in issues of feasibility and sustainability. Access to oxygen and pulse oximeters is inconsistent at Mulago, making the ViEWS difficult to operationalize in this setting. Oxygen saturations are inconsistently measured, and oxygen therapy often reflects its availability, rather than solely patient requirement. Further, although Opio et al demonstrated the predictive value of the ViEWS in an African setting, that study was conducted in a 220-bed private, not-for-profit Ugandan Catholic hospital. [18] Therefore, it is unlikely that this study reflects the feasibility challenges affecting early warning score implementation at Mulago.

The MEWS’ relative simplicity makes it attractive for use in resource-limited environments.

Additionally, the MEWS was found to have good predictive value in previous studies in Africa of patients admitted to two Tanzanian government hospitals; [17] and among medical patients in an urban emergency department in South Africa. [15]

Earlier identification of critical illness on the wards has management implications. For example, the initial steps of resuscitation for sepsis, including timely intravenous fluids, early antibiotics, and monitoring, can be delivered in resource-limited settings with potentially great impact. A before-after study of patients with septic shock in 2 emergency departments in Uganda demonstrated that early intravenous fluids, antibiotics, and routine vital sign monitoring over a 6-hour period was associated with lower 30-day mortality rates with an adjusted hazard ratio for mortality of 0.74 (CI 0.55–0.98)]. [21,27] This suggests that protocol adaptation and implementation are vital steps to addressing the burden of critical illness in this setting. Although a survey of African anesthesiologists in 2011 revealed that the Surviving Sepsis Campaign Guidelines could be implemented in entirety in less than 2% of sub-Saharan African hospitals, 75% of the Grade I recommendations could be implemented and in 67% of cases, the guidelines could be partially implemented. [11]

Timely identification of critical illness is a vital step towards establishing its overall burden in low-resource settings. This step lays the foundation to evaluate interventions that minimize morbidity and mortality. Guidelines and protocols used in high-income settings can be challenging to translate to settings with fewer resources, different patient populations, and various disease phenotypes. [9,10,28]The MEWS, a simple scoring system comprised of patients’ vital signs, is feasible for measurement at the bedside with minimal resources.

## Conclusions

The findings of this study demonstrate the need for resource-suitable, setting-specific management plans, as the proportion of ward patients with MEWS of 5 or greater will exceed the physical critical care capacity at MNRH and other low-resource hospital settings. Possible strategies include cohorting of less stable patients to enable closer monitoring and earlier intervention. Treatment of specific vital sign abnormalities with targeted therapies, including oxygen for hypoxia and intravenous crystalloid for hypotension is feasible, evidence-based, and may prevent further clinical deterioration. MEWS provides a systematic triage tool that enables identification of patients in need of such interventions, potentially preventing the need for ICU admission, and decreasing mortality.

Our prospective observational study adds to the early warning score literature arising from the Sub-Saharan African setting, and provides the first study conducted among ward patients in a fully public, government hospital. We demonstrated that the prevalence of critical illness on hospital medical and surgical wards in Mulago is substantial. Our findings suggest that routine implementation of the MEWS could be a useful triage tool to identify patients at high risk of death. Future research should include refinement of MEWS for this setting, focused MEWS education of students, ward nurses and physicians, and evaluation of available, appropriate diagnostic and therapeutic interventions based on early warning scores.

## Supporting Information

S1 Appendix Table A: Baseline characteristics of enrolled patients compared to self-discharged patients. Summary statistics are presented for baseline characteristics in the population, split by those patients we analyzed in this study (n = 452) and those who were self-discharged (n = 62); 16 of the 530 enrolled patients had missing data for MEWS or outcome at 7 days and therefore were also excluded from analysis, though they are not summarized here. Numbers are presented as N (% of available data; i.e., proportion) for categorical variables and as Mean (SD) for continuous variables. P-values are to statistically compare those patients we analyzed and those that we couldn’t due to self-discharge, and they arise from statistical tests of hypotheses: two-sample t-tests for difference in means (Age, MEWS), chi-square tests for difference in proportions (Sex, HIV status, Attendant, Admission source, HDU, Admission to Medical or Surgical, Pre-or-Post-Operative, MEWS > = 4, MEWS > = 5), and a fisher’s exact test for difference in proportions when categorical variables had expected cell counts < 5 (Admission due to trauma).
(DOCX)

S2 Appendix Table B: Univariate Analysis of Factors Associated with Mortality, with Supplemental Data. Table B represents an expanded version of Table 3, included in the manuscript. We have provided, for each binary variable within the univariate analysis, the actual proportion of patients who died and survived. This includes the variables sex, medical admission, trauma, HIV status, and MEWS divided by a cutoff of 4, and of 5. Variables age and length of stay pre-enrollment are treated as continuous variables; variable referral source is treated as categorical. Additional binary variables include the presence or absence of vital sign documentation in the ER for heart rate, blood pressure, respiratory rate, and oxygen saturation. We have included actual numbers and proportions for these variables as well.
(DOCX)
