# Supplementary material for: Modified Early Warning Score (MEWS) Identifies Critical Illness among Ward Patients in a Resource Restricted Setting in Kampala, Uganda: A Prospective Observational Study
Source: PLoS One. 2016 Mar 17;11(3):e0151408. doi: 10.1371/journal.pone.0151408 (PMC4795640; doi:10.1371/journal.pone.0151408)
Supplement: S1 Appendix — Summary statistics are presented for baseline characteristics in the population, split by those patients we analyzed in this study (n = 452) and those who were self-discharged (n = 62); 16 of the 530 enrolled patients had missing data for MEWS or outcome at 7 days and therefore were also excluded from analysis, though they are not summarized here. Numbers are presented as N (% of available data; i.e., proportion) for categorical variables and as Mean (SD) for continuous variables. P-values are to statistically compare those patients we analyzed and those that we couldn’t due to self-discharge, and they arise from statistical tests of hypotheses: two-sample t-tests for difference in means (Age, MEWS), chi-square tests for difference in proportions (Sex, HIV status, Attendant, Admission source, HDU, Admission to Medical or Surgical, Pre-or-Post-Operative, MEWS > = 4, MEWS > = 5), and a fisher’s exact test for difference in proportions when categorical variables had expected cell counts < 5 (Admission due to trauma). (DOCX) [file pone.0151408.s001.docx]

**S1 Appendix**

**Title for Table A: Baseline characteristics of enrolled patients compared to self-discharged patients***

| **Feature** |  | **Analyzed**  **(452)** | **Self-discharged**  **(62)** | **p-value for test** |
| --- | --- | --- | --- | --- |
| **Male** |  | 241 (53.5) | 28 (45.2) | 0.2278 |
| **HIV positive** |  | 110 (32.5) | 34 (69.4) | <0.0001 |
| **Attendant^*^** |  | 404 (89.4) | 54 (87.1) | 0.5884 |
| **Age (median, IQR)** |  | 42.8 (16.56) | 37.9 (15.05) | 0.0282 |
| **Admission source** | Casualty/Emergency Department | 242 (53.8) | 37 (59.7) | 0.6140 |
|  | Government unit outside Mulago | 135 (30.0) | 15 (24.2) |  |
|  | Private hospital | 73 (16.2) | 10 (16.1) |  |
| **High Dependency Unit (HDU)** |  | 4 (0.9) | 0 (0) | 0.4571 |
| **Admit due to trauma** |  | 85 (18.8) | 0 (0) | 1.0000 |
| **Admit to Medical service** |  | 204 (45.1) | 61 (98.4) | <0.0001 |
| **Admit to Surgical service** | Preoperative | 118 (54.1) | 0 (0) |  |
|  | Post operative | 100 (45.9) | 1 (100) |  |
| **MEWS (median, IQR)** |  | 2.4 (1.66) | 3.5 (1.76) | <0.0001 |
| **MEWS ≥4** |  | 96 (21.2) | 27 (43.5) | 0.0001 |
| **MEWS ≥5** |  | 53 (11.7) | 17 (27.4) | 0.0007 |

*** **Unless otherwise indicated, figures are noted as N (%)**

**Legend for Table A:** *Summary statistics are presented for baseline characteristics in the population, split by those patients we analyzed in this study (n=452) and those who were self-discharged (n=62); 16 of the 530 enrolled patients had missing data for MEWS or outcome at 7 days and therefore were also excluded from analysis, though they are not summarized here. Numbers are presented as N (% of available data; i.e., proportion) for categorical variables and as Mean (SD) for continuous variables. P-values are to statistically compare those patients we analyzed and those that we couldn’t due to self-discharge, and they arise from statistical tests of hypotheses: two-sample t-tests for difference in means (Age, MEWS), chi-square tests for difference in proportions (Sex, HIV status, Attendant, Admission source, HDU, Admission to Medical or Surgical, Pre-or-Post-Operative, MEWS >=4, MEWS >=5), and a fisher’s exact test for difference in proportions when categorical variables had expected cell counts < 5 (Admission due to trauma).*
