# Supplementary material for: Modified Early Warning Score (MEWS) Identifies Critical Illness among Ward Patients in a Resource Restricted Setting in Kampala, Uganda: A Prospective Observational Study
Source: PLoS One. 2016 Mar 17;11(3):e0151408. doi: 10.1371/journal.pone.0151408 (PMC4795640; doi:10.1371/journal.pone.0151408)
Supplement: S2 Appendix — Table B represents an expanded version of Table 3, included in the manuscript. We have provided, for each binary variable within the univariate analysis, the actual proportion of patients who died and survived. This includes the variables sex, medical admission, trauma, HIV status, and MEWS divided by a cutoff of 4, and of 5. Variables age and length of stay pre-enrollment are treated as continuous variables; variable referral source is treated as categorical. Additional binary variables include the presence or absence of vital sign documentation in the ER for heart rate, blood pressure, respiratory rate, and oxygen saturation. We have included actual numbers and proportions for these variables as well. (DOCX) [file pone.0151408.s002.docx]

**S2 Appendix**

**Table B: Univariate Analysis of Factors Associated with Mortality, with Supplemental Data**

|  |  | **Mortality** | |  | **Odds Ratio (95% CI)** | **P value** |
| --- | --- | --- | --- | --- | --- | --- |
|  |  | **Yes** | **No** | **Totals** |  |  |
| **Demographic** | Age (per year increase) | | | | 0.98 (0.95-1.01) | 0.103 |
| **features** | LOS pre-enrollment (per day increase) | | | | 0.97 (0.94-1.01) | 0.097 |
|  | Female | 11 | 200 | 211 | 0.89 (0.39-2.01) | 0.782 |
|  | Male | 14 | 227 | 241 |  |  |
|  | Totals | 25 | 427 | 452 |  |  |
|  | Medical | 22 | 182 | 204 | 9.87 (2.91-33.5) | 0.0002 |
|  | Surgical | 3 | 245 | 248 |  |  |
|  | Totals | 25 | 427 | 452 |  |  |
|  | Trauma | 3 | 82 | 85 | 0.57 (0.17-1.96) | 0.376 |
|  | Non-Trauma | 22 | 345 | 367 |  |  |
|  | Totals | 25 | 427 | 452 |  |  |
|  | HIV pos | 13 | 97 | 110 |  |  |
|  | HIV neg | 7 | 221 | 228 |  |  |
|  | Totals | 20 | 318 | 338 |  |  |
| **Patient referral** | ER |  |  |  | 0.84 (0.29-2.40) | 0.738 |
|  | District hospital |  |  |  | 0.63 (0.19-2.15) | 0.463 |
|  | Private hospital |  |  |  | 1.0 | Reference |
| **Measured vital signs** | HR measured | 14 | 180 | 194 | 1.75 (0.78-3.94) | 0.179 |
| **in ER** | HR not measured | 11 | 247 | 258 |  |  |
|  | Totals | 25 | 427 | 452 |  |  |
|  | BP measured | 17 | 178 | 195 | 2.97 (1.26-7.04) | 0.013 |
|  | BP not measured | 8 | 249 | 257 |  |  |
|  | Totals | 25 | 427 | 452 |  |  |
|  | Resp rate measured | 5 | 53 | 58 | 1.76 (0.64-4.89) | 0.276 |
|  | Resp rate not measured | 20 | 374 | 394 |  |  |
|  | Totals | 25 | 427 | 452 |  |  |
|  | 02 sat measured | 2 | 33 | 35 | 1.04 (0.24-4.6) | 0.960 |
|  | 02 sat not measured | 23 | 394 | 417 |  |  |
|  | Totals | 25 | 427 | 452 |  |  |
|  | GCS measured | 9 | 126 | 135 | 1.34 (0.58-3.12) | 0.492 |
|  | GCS not measured | 16 | 301 | 317 |  |  |
|  | Totals | 25 | 427 | 452 |  |  |
| **MEWS** | MEWS ≥ 4 | 14 | 82 | 96 | 5.35 (2.35-12.23) | <0.0001 |
|  | MEWS < 4 | 11 | 345 | 356 |  |  |
|  | Totals | 25 | 427 | 452 |  |  |
|  | MEWS ≥ 5 | 12 | 41 | 53 | 8.69 (3.72-20.29) | <0.0001 |
|  | MEWS < 5 | 13 | 386 | 399 |  |  |
|  | Totals | 25 | 427 | 452 |  |  |

**Legend for Table B***: Table 7 represents an expanded version of Table 3, included in the manuscript. We have provided, for each binary variable within the univariate analysis, the actual proportion of patients who died and survived. This includes the variables sex, medical admission, trauma, HIV status, and MEWS divided by a cutoff of 4, and of 5. Variables age and length of stay pre-enrollment are treated as continuous variables; variable referral source is treated as categorical. Additional binary variables include the presence or absence of vital sign documentation in the ER for heart rate, blood pressure, respiratory rate, and oxygen saturation. We have included actual numbers and proportions for these variables as well.*
